# Plasma Steroids as Biomarkers of Congestion and Predictors of Prognosis in Acute Heart Failure During the Vulnerable Phase

**DOI:** 10.31083/RCM53378

**Published:** 2026-07-21

**Authors:** Yangkai Fan, Jing Cui, Lu Ren, Xin Tan, Weiyao Chen, Yuan Wang

**Affiliations:** ^1^Beijing Collaborative Innovation Centre for Cardiovascular Disorders, The Key Laboratory of Remodeling-Related Cardiovascular Disease, Ministry of Education, Beijing Anzhen Hospital, Capital Medical University, 100029 Beijing, China; ^2^Beijing Institute of Heart, Lung, and Blood Vessel Diseases, 100029 Beijing, China; ^3^Department of Nutrition, The First Affiliated Hospital of Sun Yat-sen University, 510080 Guangzhou, Guangdong, China

**Keywords:** heart failure, prognosis, steroids

## Abstract

**Background:**

Patients with heart failure (HF) are at high risk of mortality and readmission during the 90-day “vulnerable phase” post-discharge, often because of persistent congestion. However, tools effective for assessing risk are lacking. Thus, this study aimed to develop a steroid-based congestion score from the plasma steroid profile and determine the associated prognostic value during the vulnerable phase in patients with acute HF.

**Methods:**

Plasma levels of 18 steroids were measured by liquid chromatography–tandem mass spectrometry in patients from the Registry Study of Biomarkers in Heart Failure. Patients were divided into a discovery cohort for biomarker screening and model development (n = 691), a cohort for validation of prognostic value (validation cohort 1, n = 267), and a cohort with paired discharge and 90-day plasma samples available for assessment of dynamic changes in biomarkers (validation cohort 2, n = 46). Steroids independently associated with the clinical congestion score (CCS) were identified by correlation analysis and multivariable logistic regression and were used to construct the “steroid-based” congestion score (SCS). The 90-day prognostic value of the SCS was assessed using Cox regression, and the incremental predictive ability of the SCS was quantified using integrated discrimination improvement.

**Results:**

The SCS included cortisol, dehydroepiandrosterone sulfate, and pregnenolone. In the discovery cohort, an SCS ≥–0.563 was associated with a 2.02-fold increase in the risk of the 90-day composite endpoint of all-cause death and HF readmission (95% confidence interval, 1.42–2.88). Adding the SCS to clinical, B-type natriuretic peptide, and clinical congestion score model increased the integrated discrimination improvement by 1.2%, 0.8%, and 1.0%, respectively (all *p* < 0.05). The prognostic value of the SCS for 90-day and long-term outcomes was confirmed in validation cohort 1. In validation cohort 2, a higher baseline SCS was associated with higher 90-day congestion rates, and SCS dynamics paralleled congestion progression.

**Conclusions:**

The SCS independently predicts adverse outcomes during the vulnerable phase in patients with HF, offering incremental prognostic value beyond traditional indicators and potentially supporting dynamic monitoring for individualized management.

## 1. Introduction

Heart failure (HF) remains a major public health challenge, imposing a significant burden on healthcare systems worldwide owing to its high mortality and readmission rates [1,2]. Studies have shown that within 60–90 days post-discharge, all-cause mortality and readmission rates can reach as high as 15% and 30%, respectively. This period is recognized as the “vulnerable phase” for patients with HF and is characterized by a peak incidence of adverse clinical outcomes [3,4,5,6].

Although in-hospital treatment can alleviate the acute symptoms of HF, many patients are discharged with persistent hemodynamic abnormalities, namely, ongoing or subclinical congestion [6,7]. This hemodynamic disturbance is considered a core pathophysiological mechanism driving death and readmission rates during the vulnerable phase [6]. Elevated left ventricular filling pressure, a hallmark of congestion, can lead directly to recurrence or worsening of symptoms such as dyspnea and edema. Therefore, accurate identification of individuals at high risk for adverse events as a result of residual hemodynamic congestion upon discharge is necessary for implementation of effective interventions during this critical window and to improve the patient’s prognosis.

However, routine clinical assessment (e.g., physical examination) has limited sensitivity for detecting subclinical congestion. While classical biomarkers such as B-type natriuretic peptide (BNP) are widely used, their interpretation can be influenced by age, renal function, and obesity; moreover, they primarily reflect myocardial wall stress and may not fully capture the complex neurohormonal and metabolic disturbances underlying congestion [8,9,10,11]. Therefore, there is a need for novel biomarkers that better reflect the hemodynamic state during the vulnerable phase in patients with HF.

Steroid hormones are a class of fat-soluble molecules that are synthesized from cholesterol through a series of enzymatic reactions and form a complex and tightly regulated network [12]. Clinical and basic research suggests that dysregulation of steroid hormone metabolism is closely linked to hemodynamic alterations in HF. In cardiometabolic risk states such as obesity and diabetes, local glucocorticoid metabolism may be altered through dysregulated steroid-converting enzymes, particularly 11β-hydroxysteroid dehydrogenase type 1 [13]. Dysregulation of mineralocorticoids (e.g., aldosterone) and glucocorticoids (e.g., cortisol) can promote sodium and water retention, increase plasma volume, thereby exacerbating cardiac load [14,15]. Furthermore, corticosteroid receptors on vascular smooth muscle can influence ion channels, mediating left ventricular systolic and diastolic dysfunction [16]. Androgens can also directly impact ventricular remodeling and function through receptors on cardiomyocytes [17]. Collectively, these mechanisms may ultimately contribute to or worsen hemodynamic deterioration in patients with HF.

We hypothesized that the plasma steroid hormone profile could better reflect the hemodynamic state during the vulnerable phase of HF than any single hormone, enabling early identification of high-risk individuals. In this study, we used a metabolomics approach to identify specific steroid hormones associated with the clinical congestion score (CCS) and constructed a steroid-based congestion score (SCS). We then evaluated the prognostic value of the SCS for all-cause mortality and readmission for HF, both individually and as a composite endpoint, within 90 days post-discharge (the vulnerable phase) in a discovery cohort and two independent validation cohorts. We also explored the incremental prognostic value of the SCS over traditional clinical parameters and BNP. Our findings indicate that the SCS is an independent and robust prognostic marker, potentially offering a novel strategy for risk stratification and personalized management during the vulnerable phase of HF.

## 2. Materials and Methods

### 2.1 Study Design and Population

This study prospectively enrolled consecutive patients hospitalized with acute heart failure (AHF) at Beijing Anzhen Hospital, Capital Medical University, from May 2017 to November 2022, who were part of the BIOMS-HF cohort (NCT03784833, https://clinicaltrials.gov/study/NCT03784833). Written informed consent was obtained from all participants prior to enrollment. The study protocol was approved by the ethics committee of Beijing Anzhen Hospital, Capital Medical University (approval number: [2016037X]), and all procedures were conducted in accordance with the Declaration of Helsinki. The study inclusion criteria were based on current guidelines for the diagnosis and management of AHF and our previously published registry cohort (BIOMS‑HF) [18,19,20]. The study inclusion criteria were as follows: age ≥18 years; diagnosis of AHF based on symptoms and signs of dyspnea at rest or on minimal exertion (orthopnea, paroxysmal nocturnal dyspnea, acute pulmonary edema), pulmonary rales, peripheral edema, and/or elevated jugular venous pressure, with pulmonary congestion confirmed by chest radiography; and plasma BNP ≥100 pg/mL or N-terminal proBNP ≥300 pg/mL. In the present study, all enrolled patients had BNP measured at admission; NT‑proBNP was not used. The patients were divided according to the phase of the study into a discovery cohort (n = 691) for biomarker screening and model development and two validation cohorts (validation cohort 1, n = 267 and validation cohort 2, n = 46) for validation of prognostic value and assessment of dynamic changes in biomarker levels, respectively. CCS was assessed at admission based on physical examination findings. Blood samples were collected after an overnight fast on the morning after admission in all cohorts. In validation cohort 2, additional blood samples were obtained at discharge and at the 90‑day follow‑up. The plasma was stored at –80 °C until analysis. Discharge CCS was recorded but was not included in the primary prognostic models, as the main analysis focused on admission CCS, it was used only to describe recongestion dynamics in validation cohort 2.

### 2.2 Clinical Congestion Score and Outcomes

The CCS was calculated based on findings on physical examination at admission. This score ranges from 0 to 3 (<1, no/mild congestion; 1 to <2, moderate congestion; ≥2, significant congestion) [21,22]. The primary outcomes were all-cause mortality and readmission for HF individually and as the composite endpoint during the 90-day vulnerable phase of HF. Readmission for HF was defined as an unplanned emergency department visit or hospitalization related to worsening HF. Patient outcomes were ascertained by telephone follow-up or from information in the electronic medical records. The median follow-up for validation cohort 1 was 376 days [interquartile range 38, 567], which allowed assessment of the prognostic value of the SCS for long-term outcomes.

### 2.3 Measurement of Steroid Hormones and Score Construction

The plasma concentrations of 18 steroid hormones were quantified using liquid chromatography–tandem mass spectrometry. The LC-MS/MS system consisted of a Waters Xevo TQ-S system (Waters Corporation, Milford, MA, USA), equipped with an ACQUITY UPLC® Peptide BEH C8 column (1.7 µm, 2.1 mm × 100 mm; Waters Corporation, Milford, MA, USA). Steroid standards (18 analytes) and internal standards (testosterone-^13^C_3_, DHEAS-d_6_, 11-deoxycortisol-d_5_, dihydrotestosterone-d_4_, and DHEA-d_5_) were purchased from Sigma (St. Louis, MO, USA). Internal standards (androstenedione-^13^C_3_, cortisol-d_4_, pregnenolone-d_4_, 17-hydroxypregnenolone-^13^C_2_-d_2_, progesterone-d_9_, estrone-d_4_, estradiol-d_4_, and aldosterone-d_8_) were obtained from Cambridge Isotope Laboratories (Tewksbury, MA, USA). Internal standards (17-hydroxyprogesterone-d_8_, cortisone-d_8_, estriol-d_3_, corticosterone-d_8_, and deoxycorticosterone-d_7_) were from Toronto Research Chemicals (Toronto, ON, Canada). For samples below the lower limit of detection, the concentration was imputed as the limit of detection value. The proportion of such imputed values for each steroid in the discovery cohort is reported in **Supplementary Table 1**. To assess the impact of this single imputation approach, we performed sensitivity analyses using four different strategies: (1) limit of detection (LOD) imputation (primary); (2) LOD/2; (3) LOD/√2; and (4) complete-case analysis (excluding all samples below the LOD). The LOD/2 and LOD/√2 methods are common ad-hoc substitution approaches that have been recommended for handling non-detects in biomarker studies [23,24]. Congestion-related steroid hormones were screened for in the discovery cohort using (1) Spearman correlation analyses of steroid hormone levels, CCS, and key clinical parameters (e.g., BNP, creatinine, heart rate) and (2) multivariable logistic regression analysis with CCS category as the dependent variable. Steroids with a Spearman *p* < 0.05 versus continuous CCS were selected for further analysis. Three hormones met this criterion: dehydroepiandrosterone sulfate (dehydroepiandrosterone sulfate (DHEAS); ρ = –0.13, *p* = 0.001), pregnenolone (ρ = 0.12, *p* = 0.001), and cortisol (ρ = 0.11, *p* = 0.004). The level of each hormone was normalized to the Z-score. The weight of association (regression coefficient) of each hormone with congestion status (CCS ≥1) was obtained from the logistic regression model. The SCS was calculated using the following formula:

SCS = –0.7716 + (0.2686 × Z_cortisol_) – (0.2004 × Z_DHEAS_) – (0.0131 × Z_pregnenolone_).

### 2.4 Statistical Analysis

Continuous variables are expressed as the mean ± standard deviation or median [interquartile range] and were compared between cohorts using analysis of variance or the Kruskal–Wallis test. Categorical variables are shown as the frequency (percentage) and were compared between cohorts using the chi-squared test. To evaluate the prognostic value of the SCS, we used Cox proportional hazards regression models to calculate hazard ratios (HRs) and 95% confidence intervals (CIs) for 90-day outcomes, with the SCS treated as a continuous variable or categorized by its optimal cut-off value. The optimal cut-off value was determined using “maximally selected rank statistics” based on its performance as a predictor of the 90-day composite endpoint [25]. Kaplan–Meier curves were generated, and differences in survival were examined using the log-rank test. Four baseline models were constructed to assess the incremental prognostic value of the SCS: (1) a clinical model using variables selected by stepwise regression; (2) a BNP model comprising log-transformed BNP only; (3) a CCS model based on CCS category; and (4) a high-sensitivity troponin I (hs-TnI) model. For the baseline clinical model, variable selection was performed using akaike information criterion (AIC)-based bidirectional stepwise regression from a candidate variable pool. The final model included the following covariates: age, male sex, systolic blood pressure (SBP), heart rate, body mass index (BMI), creatinine (Cr), angiotensin-converting enzyme (ACE) inhibitor/angiotensin receptor blocker (ARB) use, and diuretic use. Improvement in model performance upon adding the SCS was quantified by changes in the area under the curve, net reclassification improvement, and integrated discrimination improvement (IDI). Subgroup analyses of factors that included age, sex, comorbidities, left ventricular ejection fraction (LVEF), and use of diuretics were performed in pre-defined subgroups, and interactions between the SCS and these subgroups were tested. Decision curve analysis was used to evaluate the net clinical benefit of the SCS. To assess the risk of overfitting and correct for optimism, we performed internal validation within the discovery cohort, including 500 iterations of bootstrap resampling and 10‑fold cross‑validation. In each bootstrap iteration, the optimal cut‑off was re‑estimated using maximally selected rank statistics, and the 95% confidence interval was derived from the bootstrap distribution. For 10‑fold cross‑validation, the discovery cohort was randomly split into 10 equal folds; in each round, 9 folds were used for model training and the remaining fold for evaluating out‑of‑fold log‑rank *p* values and C‑index. Calibration was assessed by plotting predicted versus observed event probabilities, with optimism correction using bootstrap. The optimism‑corrected C‑index was calculated as the apparent C‑index minus the mean optimism estimated from 500 bootstrap resamples. All statistical analyses were performed using R (version 4.0.2; R Foundation for Statistical Computing, Vienna, Austria). All tests were two-sided, and a *p*-value of < 0.05 was considered statistically significant.

## 3. Results

### 3.1 Baseline Characteristics

**Fig. 1.**
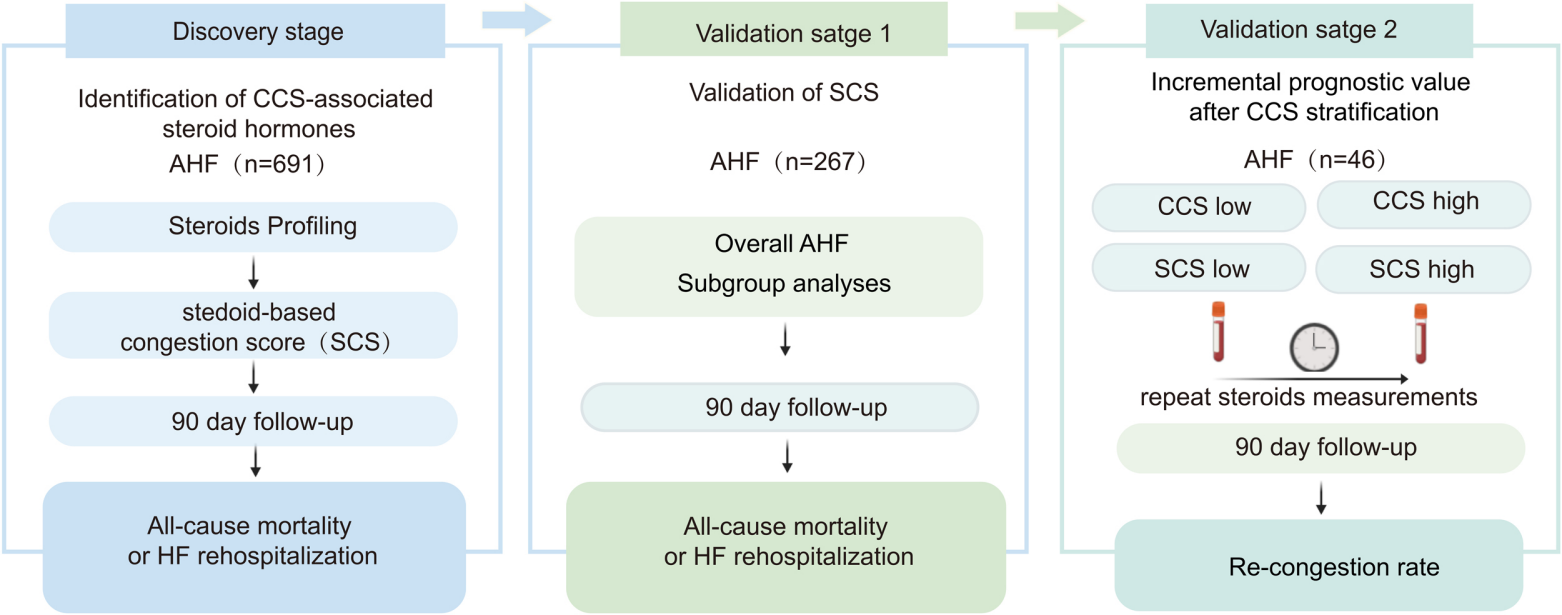
**Study design and flow chart**. The study was divided into three stages. The first was a discovery stage (n = 691), in which steroid hormones associated with the clinical congestion score (CCS) were identified and used to construct the steroid-based congestion score (SCS). The second was a validation stage 1 (n = 267), in which the prognostic value of the SCS for 90-day and long-term outcomes was evaluated. The third was a validation stage 2 (n = 46), during which the association between the SCS at baseline and the recongestion rate at 90 days was assessed by repeated steroid measurements. AHF, acute heart failure.

The study flow diagram is shown in Fig. 1. The 691 patients with AHF in the discovery cohort were categorized by CCS into three groups: 0 ≤ CCS < 1 (no/mild congestion, n = 470), 1 ≤ CCS < 2 (moderate congestion, n = 195), and CCS ≥2 (significant congestion, n = 26). Higher CCS grades were associated with significantly greater severity of HF and characterized by higher proportions of New York Heart Association functional class III/IV, higher BNP, more prevalent signs and symptoms of congestion (orthopnea, edema, hepatomegaly, ascites, elevated jugular venous pressure), worse renal function (higher creatinine, lower estimated glomerular filtration rate), and more pronounced electrolyte imbalance, including hyponatremia and hyperkalemia (all *p* < 0.05, Table 1). Baseline medications at admission in the discovery cohort are also summarized in Table 1; the most commonly prescribed drugs were diuretics (70.8%), β‑blockers (61.9%), spironolactone (50.4%), and ACEi/ARB (26.6%).

**Table 1.** **Baseline clinical characteristics in the discovery cohort according to the clinical congestion score**.

		No/mild congestion 0 ≤ CCS < 1 (n = 470)	Moderate congestion 1 ≤ CCS <2 (n = 195)	Significant congestion CCS ≥2 (n = 26)	*p* value
Demographics					
	Age, years	62.5 (14.2)	63.1 (14.3)	59.8 (15.5)	0.532
	Male sex (n, %)	300 (63.8%)	131 (67.2%)	17 (65.4%)	0.711
	BMI, kg/m^2^	24.4 (5.81)	25.2 (5.54)	25.2 (4.39)	0.305
	NYHA class III/IV (n, %)	314 (66.8%)	168 (86.2%)	25 (96.2%)	<0.001
	Smoking (n, %)	115 (24.5%)	64 (32.8%)	3 (11.5%)	0.018
Vital signs					
	Heart rate, beats/min	80.8 (18.8)	83.9 (18.6)	82.5 (18.3)	0.146
	SBP, mm Hg	123 (20.1)	124 (21.5)	129 (21.2)	0.304
	DBP, mm Hg	72.7 (13.2)	74.4 (14.3)	71.4 (12.0)	0.273
Clinical profile (n, %)					
	Orthopnea	0 (0.00%)	162 (83.1%)	23 (88.5%)	<0.001
	Oedema	76 (16.2%)	72 (36.9%)	22 (84.6%)	<0.001
	Cardiac murmur	137 (29.1%)	58 (29.7%)	11 (42.3%)	0.361
	Hepatomegaly	16 (3.40%)	14 (7.18%)	6 (23.1%)	<0.001
	Ascites	7 (1.49%)	6 (3.08%)	3 (11.5%)	0.009
	Rales	113 (24.0%)	95 (48.7%)	13 (50.0%)	<0.001
	Jugular venous pressure	0 (0.00%)	16 (8.21%)	13 (50.0%)	<0.001
Echocardiography					
	LVEF, %	46.5 (14.8)	41.3 (14.1)	46.0 (15.7)	<0.001
	E/A	1.42 (1.18)	1.62 (1.19)	1.69 (1.13)	0.099
	IVST, mm	10.1 (3.99)	10.2 (2.63)	11.7 (12.0)	0.195
	LVEDD, mm	55.7 (10.8)	58.4 (11.7)	55.3 (9.81)	0.015
	LVESD, mm	41.7 (12.2)	45.4 (13.2)	41.0 (11.0)	0.002
Laboratory					
	BNP, pg/mL	566 [330; 1088]	833 [439; 1518]	881 [569; 1632]	<0.001
	Hemoglobin, g/L	131 (24.0)	131 (23.3)	119 (27.8)	0.05
	HDL, mmol/L	1.09 (0.72)	1.02 (0.77)	1.04 (0.39)	0.499
	LDL, mmol/L	2.50 (0.87)	2.44 (0.87)	2.29 (0.76)	0.38
	Na, mmol/L	139 (3.85)	138 (4.56)	137 (5.71)	0.001
	K, mmol/L	4.21 (0.53)	4.22 (0.62)	4.58 (0.63)	0.005
	Cr, µmol/L	83.6 [67.7; 113]	99.7 [76.8; 135]	99.1 [79.6; 206]	<0.001
Medical history (n, %)					
	Diabetes	146 (31.1%)	83 (42.6%)	7 (26.9%)	0.013
	Hypertension	249 (53.0%)	117 (60.0%)	16 (61.5%)	0.204
	Hyperlipidemia	172 (36.6%)	83 (42.6%)	10 (38.5%)	0.354
	Coronary artery disease	230 (48.9%)	105 (53.8%)	14 (53.8%)	0.484
	Valvular disease	220 (46.8%)	78 (40.0%)	9 (34.6%)	0.162
Baseline medications (n, %)					
	ACEi/ARB	123 (26.2%)	54 (27.7%)	7 (26.9%)	0.921
	Beta-blockers	286 (60.9%)	126 (64.6%)	16 (61.5%)	0.66
	Spironolactone	232 (49.4%)	108 (55.4%)	8 (30.8%)	0.046
	Diuretics	337 (71.7%)	134 (68.7%)	18 (69.2%)	0.732
	Digoxin	116 (24.7%)	53 (27.2%)	6 (23.1%)	0.768
	ARNI	65 (13.8%)	39 (20.0%)	3 (11.5%)	0.122
Steroids(ng/mL)					
	Estrone	0.005 [0.005; 0.054]	0.005 [0.005; 0.063]	0.005 [0.005; 0.062]	0.38
	Estradiol	0.02 [0.01; 0.03]	0.02 [0.01; 0.03]	0.01 [0.01; 0.02]	0.544
	Estriol	0.005 [0.005; 0.009]	0.005 [0.005; 0.010]	0.005 [0.005; 0.020]	0.059
	17-hydroxypregnenolone	0.44 [0.23; 1.13]	0.48 [0.20; 1.23]	0.27 [0.19; 0.51]	0.168
	Dehydroepiandrosterone	0.98 [0.51; 2.10]	0.97 [0.48; 1.92]	0.49 [0.38; 1.25]	0.098
	Androstenedione	0.53 [0.34; 0.86]	0.63 [0.40; 1.11]	0.49 [0.30; 0.78]	0.011
	Testosterone	1.27 [0.18; 3.30]	1.84 [0.25; 3.59]	0.68 [0.15; 2.97]	0.054
	Dihydrotestosterone	0.18 [0.06; 0.38]	0.18 [0.07; 0.38]	0.14 [0.03; 0.44]	0.908
	Pregnenolone	1.43 [0.51; 4.19]	2.10 [0.72; 5.88]	1.00 [0.37; 4.23]	0.003
	Progesterone	0.06 [0.04; 0.12]	0.08 [0.05; 0.15]	0.09 [0.04; 0.15]	0.16
	11-deoxycorticosterone	0.04 [0.02; 0.09]	0.04 [0.03; 0.10]	0.05 [0.02; 0.08]	0.667
	17-hydroxyprogesterone	0.50 [0.26; 0.88]	0.57 [0.28; 0.89]	0.49 [0.18; 1.01]	0.744
	Corticosterone	2.46 [1.11; 5.05]	2.58 [1.17; 5.97]	3.04 [1.27; 4.76]	0.727
	11-deoxycortisol	0.24 [0.11; 0.55]	0.23 [0.12; 0.70]	0.18 [0.12; 0.40]	0.569
	Cortisone	16.7 [12.4; 20.5]	16.5 [11.6; 21.6]	17.1 [13.0; 22.1]	0.966
	Cortisol	145 [103; 211]	176 [118; 261]	189 [114; 245]	0.003
	DHEAS	704 [302; 1410]	652 [292; 1230]	372 [116; 572]	0.001
	Aldosterone	0.08 [0.03; 0.21]	0.07 [0.03; 0.22]	0.08 [0.03; 0.21]	0.92

Abbreviations: ACEi, angiotensin-converting enzyme inhibitor; ARB, angiotensin receptor blocker; ARNI, angiotensin receptor–neprilysin inhibitor; BMI, body mass index; BNP, B-type natriuretic peptide; Cr, creatinine; DBP, diastolic blood pressure; DHEAS, dehydroepiandrosterone sulfate; E/A, E/A ratio; HDL, high-density lipoprotein; LVEF, left ventricular ejection fraction; IVST, interventricular septal thickness; LVEDD, left ventricular end-diastolic diameter; LVESD, left ventricular end-systolic diameter; LDL, low-density lipoprotein; NYHA, New York Heart Association; SBP, systolic blood pressure; CCS, clinical congestion score.

Among the 18 steroids measured in plasma, cortisol levels increased significantly with higher CCS, while DHEAS levels decreased significantly (*p* < 0.05), suggesting a monotonic relationship between these two hormones and severity of congestion. Notably, pregnenolone levels showed a non-linear pattern, increasing from 1.43 ng/mL to 2.10 ng/mL in the group with no/mild or moderate congestion (CCS <2) and decreasing to 1.00 ng/mL in the group with significant congestion (CCS ≥2). This initial increase followed by a decrease suggests that pregnenolone, as a precursor for all steroid hormones, may be elevated in the early stages of congestion in a compensatory manner but becomes depleted as a result of synthetic exhaustion when congestion becomes severe.

### 3.2 Steroid Hormones Associated with the Clinical Congestion Score

**Fig. 2.**
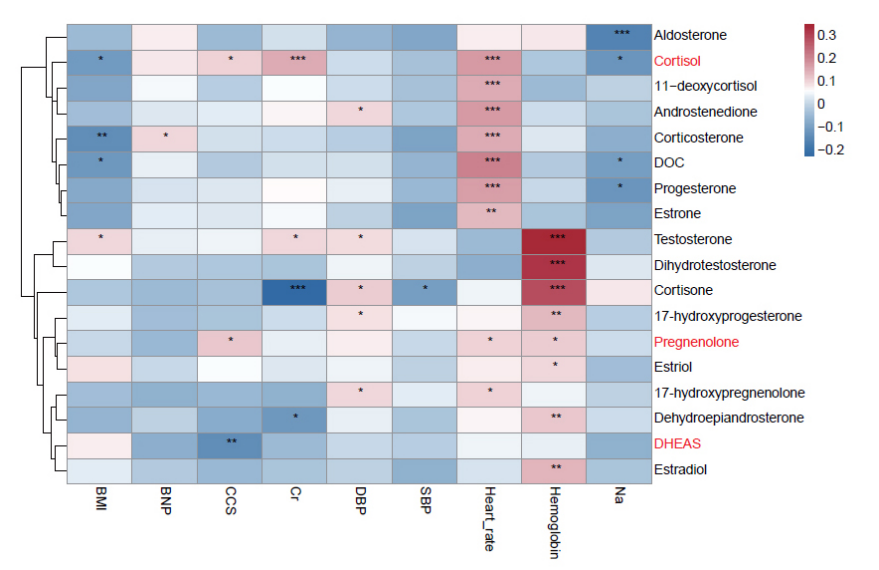
**Correlations of steroid hormones with the clinical congestion score and key clinical parameters in the discovery cohort**. Heatmap showing Spearman correlation coefficients for plasma steroid hormones (rows) and selected clinical variables (columns), including the CCS, BNP, Cr, BMI, blood pressure, heart rate, hemoglobin, and sodium. The color scale represents the magnitude and direction of correlation. Asterisks indicate statistical significance (*p < 0.05, **p < 0.01, ***p < 0.001). Steroid hormones that were significantly correlated with the CCS (cortisol, DHEAS, and pregnenolone) are in red text. BMI, body mass index; BNP, B-type natriuretic peptide; CCS, clinical congestion score; Cr, creatinine; DBP, diastolic blood pressure; DHEAS, dehydroepiandrosterone sulfate; DOC, 11-deoxycorticosterone; SBP, systolic blood pressure.

Correlation analysis in the discovery cohort revealed that several steroid hormones were significantly associated with the CCS (Fig. 2). As shown in **Supplementary Table 1**, the three steroids comprising the SCS had very low proportions of values below the LOD (cortisol 0.72%, DHEAS 0.43%, pregnenolone 0%). Notably, cortisol showed a significant positive correlation with the CCS (r = 0.11, *p* = 0.004) and the DHEAS demonstrated a significant negative correlation (r = –0.13, *p* = 0.001). These findings are consistent with the monotonic trends observed in Table 1. Pregnenolone showed a significant positive correlation with the CCS (r = 0.12, *p* = 0.001), primarily reflecting the progression from no/mild to moderate congestion; however, the decrease in the significant congestion group may not have been fully captured in the linear correlation analysis because of the small sample size (n = 26). There was a potential non-linear relationship between pregnenolone and the severity of congestion, consistent with the pattern of an initial increase followed by a decrease observed in Table 1.

Notably, aldosterone showed a weak correlation with CCS that was not statistically insignificant. From the perspective of the steroid metabolic network, the hormones significantly associated with CCS, namely, pregnenolone (a synthetic precursor), cortisol (a glucocorticoid), and DHEAS (an androgen precursor), represent key nodes in different branches of the steroidogenesis pathway. This finding suggested that metabolic reprogramming of steroids in the congestive state may involve a dynamic imbalance between the synthetic reserve upstream and the stress/sex hormone axes downstream.

Furthermore, the levels of these CCS-related hormones correlated consistently with the values for other markers of severity of HF. For example, the cortisol level correlated positively with heart rate (r = 0.18, *p* < 0.001) and negatively with serum sodium (r = –0.11, *p* = 0.003), pregnenolone correlated positively with hemoglobin (r = 0.33, *p* < 0.001), and DHEAS correlated positively with both heart rate (r = 0.22, *p* < 0.001) and hemoglobin (r = 0.36, *p* < 0.001) (Fig. 2, **Supplementary Table 2**). BNP showed weak correlations with most of the steroid hormones, indicating that steroid levels may provide pathophysiological information distinct from BNP.

### 3.3 Prognostic Value of the SCS for 90-Day Outcomes

After 90 days of follow-up, 161 (23.3%) composite endpoint events occurred in the discovery cohort (73 deaths, 98 readmissions for HF). Using maximally selected rank statistics, the SCS had an optimal cut-off value of –0.563 for prediction of the 90-day composite endpoint.

**Fig. 3.**
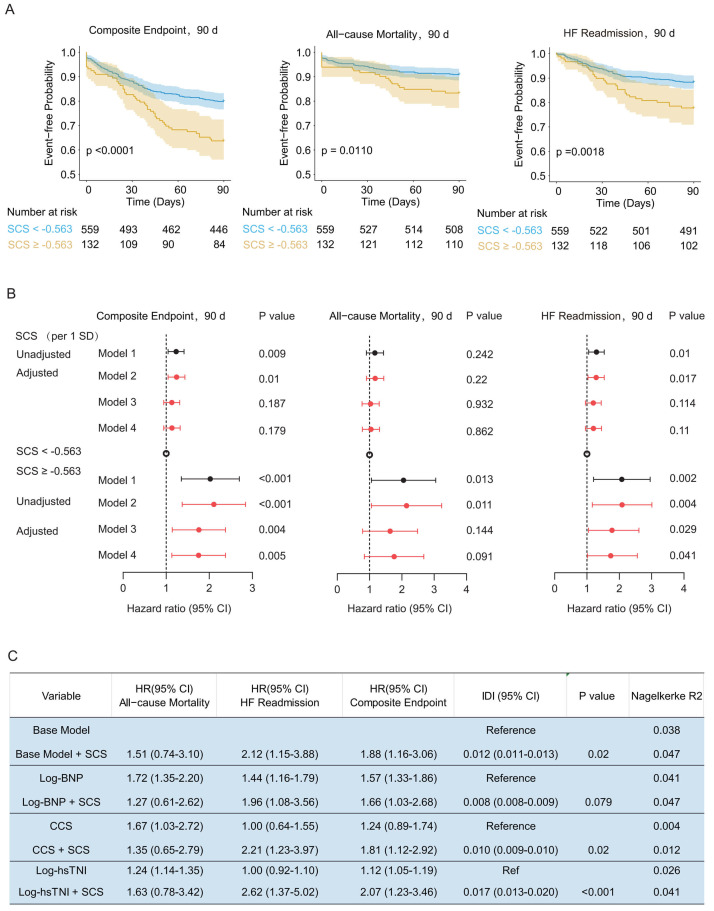
**Prognostic value of the steroid-based congestion score for 90-day outcomes in the discovery cohort**. (A) Kaplan–Meier curves for the composite endpoint (all-cause mortality and readmission for HF) according to SCS category (low, SCS <–0.563; high, SCS ≥–0.563). (B) Multivariable Cox regression for 90-day outcomes in the discovery cohort. Model 1: unadjusted. Model 2: adjusted for age and sex. Model 3: adjusted for age, sex, log-transformed BNP, eGFR, sodium, LVEF, NYHA class III/IV, diabetes, loop diuretics, ACEi/ARB, beta-blockers, and spironolactone. Model 4: further adjusted for coronary artery disease, valvular disease, and ARNI. (C) Table showing the increase in risk (i.e., an increase in the HR) for the composite endpoint, all-cause mortality, and readmission for HF, along with improvements in the IDI and Nagelkerke R^2^ values when the SCS is added to the baseline clinical model, BNP model, CCS model and hs-TnI model. BNP, B-type natriuretic peptide; CCS, clinical congestion score; CI, confidence interval; HF, heart failure; HR, hazard ratio; IDI, integrated discrimination improvement; SCS, steroid-based congestion score; hs-TnI, high-sensitivity troponin I; SD, standard deviation; eGFR, estimated glomerular filtration rate.

Kaplan–Meier analysis showed that the cumulative incidences of 90-day all-cause mortality, readmission for HF, and the composite endpoint were significantly higher in patients with an SCS of ≥–0.563 than in those with an SCS of <–0.563 (Model 1, *p* < 0.05, log-rank test, Fig. 3A). Multivariable Cox regression confirmed that the SCS was an independent predictor of adverse 90-day outcomes, both as a continuous variable and as a categorical variable. After adjusting for age and sex, patients in the high SCS group (≥–0.563) had a 2.02-fold higher risk of the composite endpoint (Model 2, 95% CI: 1.42–2.88, *p* < 0.001), a 1.96-fold higher risk of all-cause mortality (Model 2, 95% CI: 1.16–3.31, *p* = 0.011), and a 1.95-fold higher risk of readmission for HF (Model 2, 95% CI: 1.24–3.07, *p* = 0.004) than those in the low SCS group. Multivariable Cox regression with progressive adjustment demonstrated that the high SCS group remained independently associated with the composite endpoint after adjustment for established HF risk factors (Model 3: HR = 1.69, 95% CI: 1.18–2.41, *p* = 0.004; Model 4: HR = 1.68, 95% CI: 1.17–2.41, *p* = 0.005). For HF rehospitalization, the high SCS group also remained significant in adjusted models (Model 3: HR = 1.66, 95% CI: 1.05–2.61, *p* = 0.029; Model 4: HR = 1.62, 95% CI: 1.02–2.56, *p* = 0.041). However, for all-cause mortality, the association was no longer significant after full adjustment (Model 3: HR = 1.49, 95% CI: 0.87–2.55, *p* = 0.144; Model 4: HR = 1.60, 95% CI: 0.93–2.75, *p* = 0.091), possibly reflecting the limited number of deaths (n = 73) or a closer link between SCS and HF rehospitalization (Fig. 3B, **Supplementary Table 3**). Internal validation via bootstrap and 10‑fold cross‑validation confirmed the stability of the cut‑off (**Supplementary Fig. 1A, B**) but showed limited reproducibility of the prognostic signal across subsamples (**Supplementary Fig. 1C**). Calibration was good in apparent analysis (**Supplementary Fig. 1D**) and after bootstrap correction (**Supplementary Fig. 1E**), although out‑of‑fold calibration indicated mild overfitting (**Supplementary Fig. 1F**). The apparent C‑index of 0.680 was optimism‑corrected to 0.649 (**Supplementary Fig. 1G**), suggesting genuine but modestly optimistic prognostic value.

### 3.4 Incremental Predictive Value of the SCS

Fig. 3C demonstrates the incremental predictive value of the SCS beyond the four baseline models. Adding SCS to the baseline clinical model (which includes multiple clinical risk factors) increased the risk of 90-day all-cause mortality, readmission for HF, and the composite endpoint by 51%, 112%, and 88%, respectively, with a significant improvement in the IDI value of 1.2% (*p* < 0.05) and an increase in the Nagelkerke R^2^ value from 0.038 to 0.047. Adding SCS to the BNP model increased the risk of readmission for HF and the composite endpoint by 96% and 66%, respectively, with a significant increase in the IDI value of 0.8% (*p* < 0.05). Addition of the SCS to the CCS model increased the risk of readmission for HF and the composite endpoint by 121% and 81%, respectively, with a significant increase in the IDI value of 1.0% (*p* < 0.05). Furthermore, adding SCS to the hs-TnI model increased the risk of the 90-day composite endpoint by 162%, with an IDI of 0.017 (95% CI: 0.013–0.020, *p* < 0.001) and an increase in Nagelkerke R^2^ from 0.026 to 0.041. Correlation analysis showed a very weak correlation between SCS and log-hs-TnI (Spearman’s ρ = 0.004) and a weak correlation between SCS and log-BNP (ρ = 0.114), whereas hs-TnI and BNP had a modest correlation (ρ = 0.211). These results indicated that the SCS provides incremental prognostic information beyond traditional clinical factors, BNP, and physical examination.

### 3.5 Validation of the Prognostic Value of the SCS

**Fig. 4.**
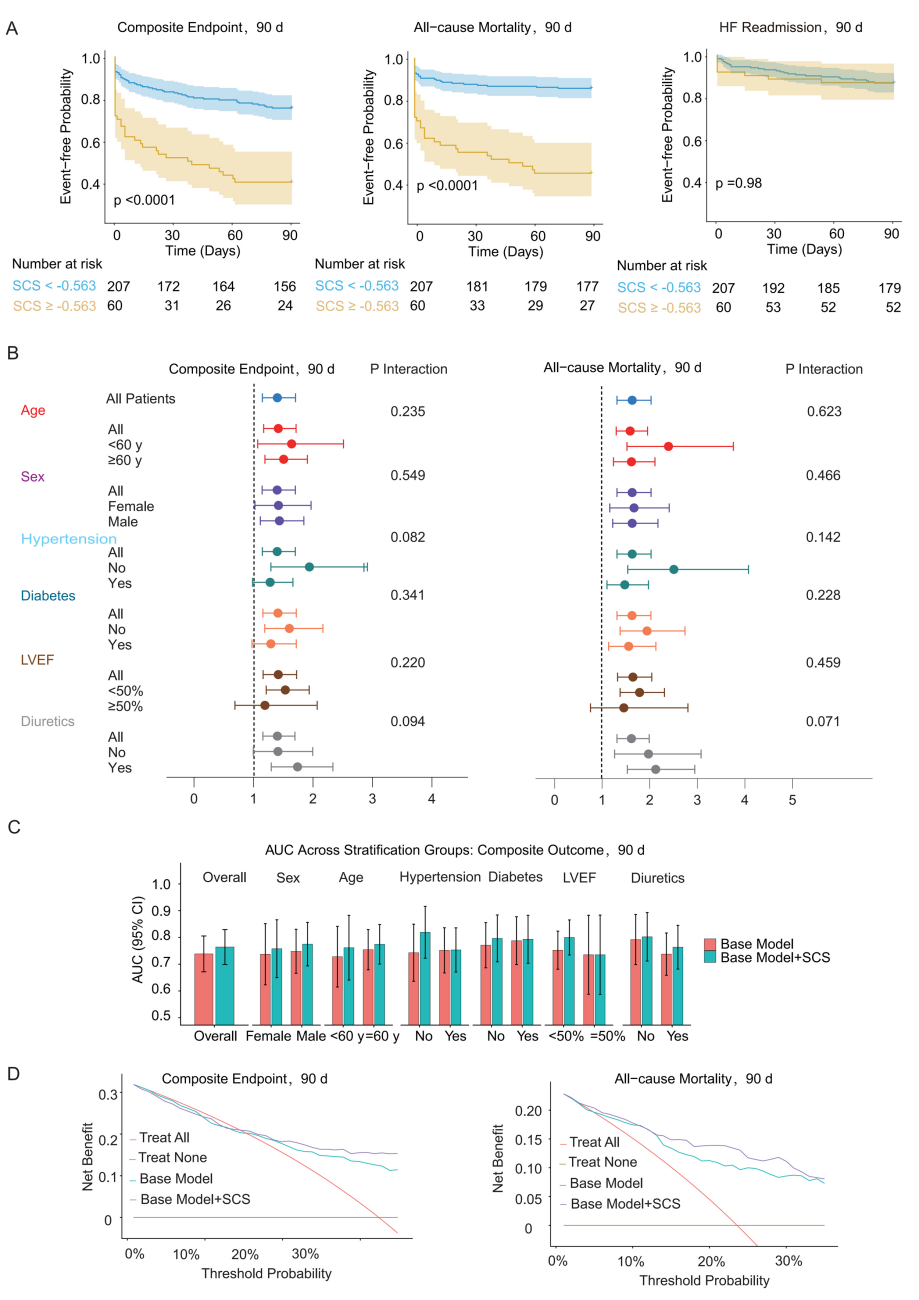
**Validation of the prognostic value of the SCS for 90-day outcomes in validation cohort 1**. (A) Kaplan–Meier curves for the composite endpoint according to SCS category. (B) Subgroup analysis for the composite endpoint: forest plot showing hazard ratios (high vs. low SCS) across subgroups based on age, sex, hypertension, diabetes, LVEF, and use of diuretics. *p*-values for interaction are shown. (C) Discrimination performance (AUC) of the baseline model and the baseline model plus the SCS for the composite endpoint in the overall population and across subgroups. (D) Decision curve analysis for the composite endpoint, comparing the net clinical benefit of the clinical baseline model with and without the SCS. AUC, area under the curve; CI, confidence interval; HF, heart failure; LVEF, left ventricular ejection fraction; SCS, steroid-based congestion score.

These findings were successfully validated in validation cohort 1 (n = 267). During the 90-day follow-up, 87 (32.6%) composite endpoint events occurred. As in the discovery cohort, patients with an SCS of ≥–0.563 had significantly higher risks of the 90-day composite endpoint (HR 1.39, 95% CI 1.14–1.70) and all-cause mortality (HR 1.64, 95% CI 1.32–2.04) than those with a low SCS (Fig. 4A, **Supplementary Table 4**). Subgroup analysis confirmed that the prognostic value of the SCS was consistent when stratified by age, sex, hypertension, and diabetes (*p*_interaction_ > 0.05 for all) (Fig. 4B, **Supplementary Table 4**).

Decision curve analysis demonstrated that addition of the SCS to the baseline clinical model was associated with a greater net clinical benefit across a wide range of threshold probabilities when compared with the baseline clinical model alone (Fig. 4C). Furthermore, incorporating the SCS into the baseline clinical model significantly improved the discriminative ability of the model for the 90-day composite endpoint, with the area under the curve increasing from 0.73 (95% CI 0.67–0.81) to 0.76 (95% CI 0.70–0.83) and a significant IDI value (*p* < 0.05). Exploratory subgroup analyses suggested that this improvement was consistent across subgroups of sex, age, hypertension, diabetes, LVEF, and diuretic use (Fig. 4D, **Supplementary Table 4**); however, the absence of statistically significant interactions does not confirm homogeneous effects across all subgroups, particularly given the limited sample size in some strata.

As shown in **Supplementary Fig. 2** and **Supplementary Table 5**, all four imputation strategies yielded nearly identical effect estimates. In the discovery cohort, the HR (high vs. low) for the 90-day composite endpoint was 1.69 (95% CI: 1.18–2.41) for LOD, LOD/2, and LOD/√2, and 1.75 (95% CI: 1.22–2.51) for complete-case analysis. Similarly consistent results were observed in validation cohort 1. These findings confirm that the single LOD imputation used in the primary analysis did not materially affect the robustness of the SCS.

### 3.6 Prognostic Value of the SCS for Long-Term Outcomes

**Fig. 5.**
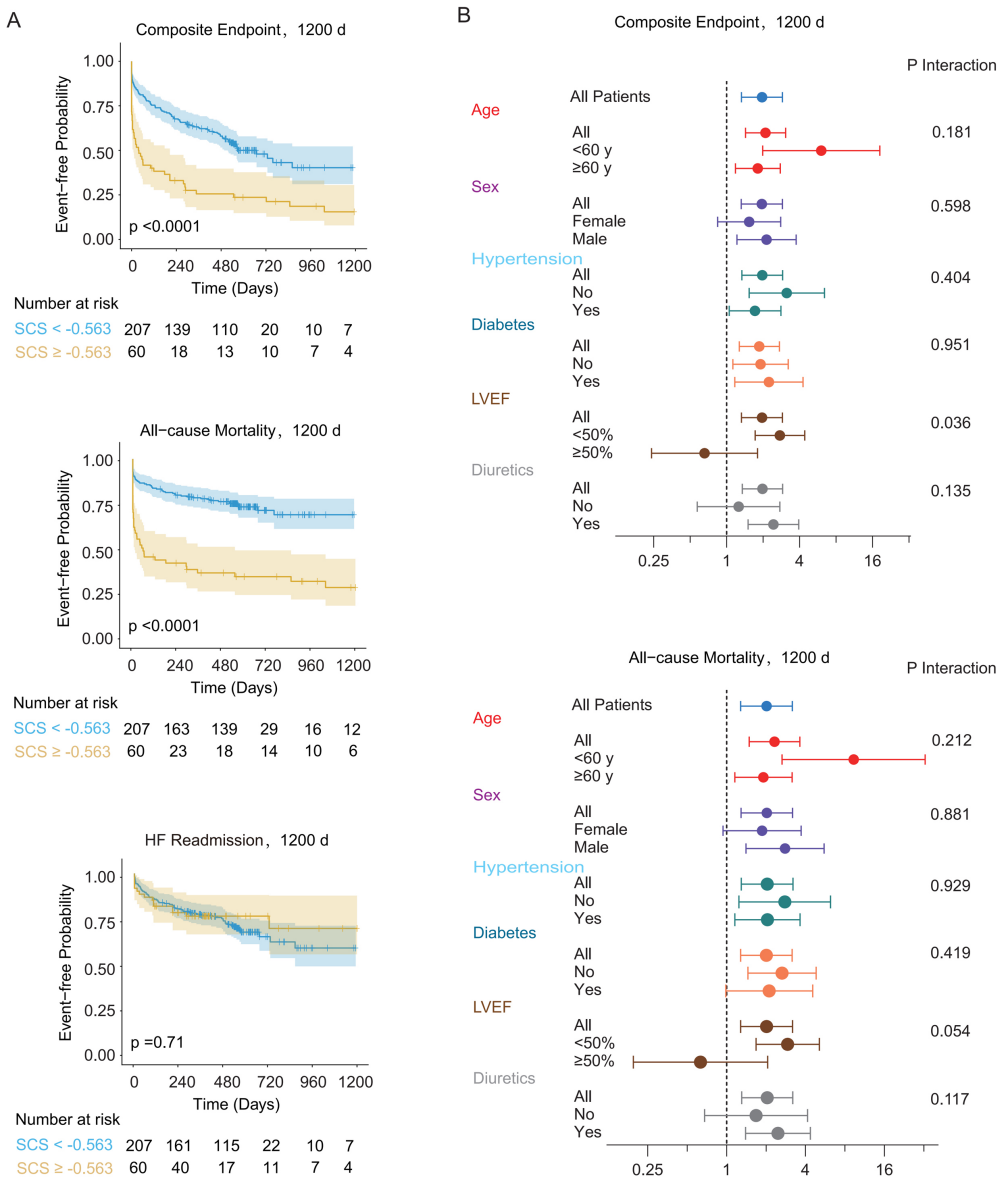
**Prognostic value of SCS for long-term outcomes in validation cohort 1**. (A) Kaplan–Meier curves for the composite endpoint, all-cause mortality, and readmission for HF. (B) Subgroup analysis for the composite endpoint and all-cause mortality, with forest plots showing hazard ratios (high vs. low SCS) across subgroups. LVEF, left ventricular ejection fraction; SCS, steroid-based congestion score.

In validation cohort 1, during a median follow-up of 376 days [interquartile range 38, 567], 151 (56.5%) composite endpoint events occurred (97 deaths, 79 readmissions for HF). The SCS also demonstrated strong prediction performance for long-term outcomes: the risk of the long-term composite endpoint was nearly double in the group with a high (≥–0.563) SCS (HR 1.96, 95% CI 1.33–2.90), as were the risks of all-cause mortality (HR 2.02, 95% CI 1.28–3.18), and readmission for HF (HR 1.96, 95% CI 1.24–3.10) (Fig. 5A, **Supplementary Table 6**).

Subgroup analyses revealed higher HR estimates for long-term all-cause mortality in men, patients with an LVEF <50%, those using diuretics, and those without diabetes. However, all *p*-values for interaction were >0.05 (range 0.054–0.419), indicating no statistically significant differences in the prediction performance of the SCS across subgroups according to age, sex, hypertension, diabetes, LVEF, and use of diuretics. These findings indicated that the prognostic value of the SCS for long-term outcomes remained largely consistent across subgroups (Fig. 5B, **Supplementary Table 6**).

**Fig. 6.**
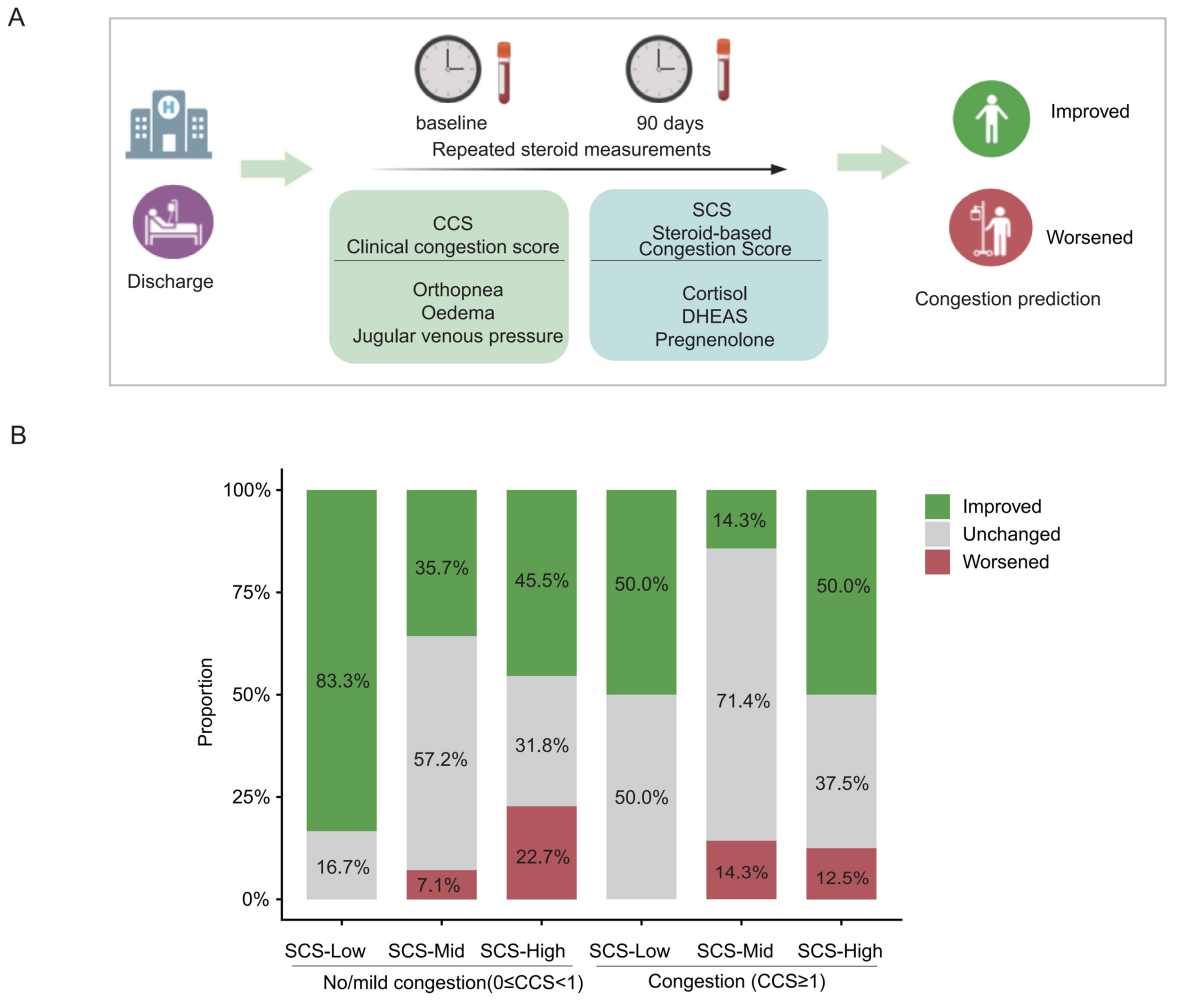
**Association of baseline SCS with the recongestion rate at 90 days in validation cohort 2**. (A) Schematic diagram showing the study design for validation cohort 2, including collection of blood samples at discharge and at the 90-day follow-up and measurement of steroid hormones by liquid chromatography–tandem mass spectrometry. (B) Bar graphs showing the proportion of patients who had developed congestion (CCS ≥1) by the 90-day follow-up when stratified by SCS tertile at baseline. CCS, clinical congestion score; SCS, steroid-based congestion score.

We also analyzed the prediction performance of the SCS in validation cohort 2 (n = 46) based on tertile for the risk of congestion during the vulnerable phase (Fig. 6). Among patients with no/mild congestion at discharge (0 ≤ CCS < 1), those in the second and third SCS tertiles had a 7.1% and 22.7% greater likelihood of experiencing congestion (CCS ≥1) during the 90-day follow-up, respectively, than those in the first SCS tertile. Among patients with moderate or worse congestion at discharge (CCS ≥1), those in the second and third SCS tertiles had a 14.3% and 12.5% greater likelihood of persistent or recurrent congestion, respectively, than those in the first SCS tertile. Medication use in this cohort was recorded descriptively: at discharge, β-blockers, spironolactone, and diuretics were used in 65.2%, 52.2%, and 52.2% of patients, respectively; at 3-month follow-up, the corresponding rates were 58.7%, 50.0%, and 63.0%. Given the small sample size (n = 46), these findings should be considered exploratory. The effect estimates were imprecise, with wide confidence intervals (e.g., OR for SCS high vs. low = 1.57, 95% CI: 0.07–35.03; **Supplementary Table 7**). Although a trend toward higher baseline SCS associated with greater recongestion risk was observed, these exploratory analyses do not provide confirmatory evidence and require validation in larger cohorts.

## 4. Discussion

In this study, we systematically profiled plasma steroid hormone levels in patients with AHF and constructed and validated a novel congestion score, the SCS, based on three hormones: cortisol, DHEAS, and pregnenolone. Our main findings can be summarized as follows: (1) in patients with AHF, the steroid hormone profile, particularly the stress and protective hormonal axes represented by pregnenolone as a synthetic precursor and cortisol and DHEAS as downstream effectors, is closely associated with clinical congestion status; (2) the SCS derived from these hormones is an independent and robust predictor of 90-day (vulnerable-phase) and long-term all-cause mortality and readmission for HF; and (3) the SCS not only provides incremental prognostic information beyond traditional clinical risk factors, BNP, and physical examination, but also demonstrates robust performance across multiple subgroups and independent internal validation cohorts, with favorable net clinical benefit. These findings suggest that the multi-hormone SCS could serve as a novel tool for assessment of the congestion burden and risk stratification in HF, particularly for identifying high-risk patients during the critical vulnerable phase post-discharge. Given the observational nature of this study, the reported associations should not be interpreted as causal, and the mechanistic interpretations proposed herein are speculative and hypothesis-generating, requiring confirmation in future experimental and translational studies.

The composition of the SCS and its underlying biological rationale are central to this study. Our results showed that the SCS is jointly constituted by cortisol (with a positive coefficient as a risk factor) and DHEAS/pregnenolone (with negative coefficients as protective factors). This combination may reflect an imbalance between catabolic (stress) and anabolic (protective) hormonal axes in patients with HF, although this interpretation remains speculative [26]. Cortisol is a classic stress hormone; in the setting of HF-related neurohormonal activation, elevated cortisol may contribute to hemodynamic compromise and ventricular remodeling through volume retention, increased vascular resistance/afterload, and myocardial fibrosis [27,28,29]. DHEAS is the most abundant circulating steroid and has potential protective effects, including vasodilatory, antioxidant, and immunomodulatory properties. A low DHEAS level has been associated with a poor prognosis in patients with HF [30,31,32,33]. Pregnenolone is the precursor for all steroid hormones, and its decline in patients with HF and congestion may reflect overall impairment of synthesis of anabolic steroids or a shift towards production of stress hormones [34]. Therefore, by integrating these three hormones, which represent stress (cortisol), protection (DHEAS), and synthetic reserve (pregnenolone), the SCS captures the complex neurohormonal and metabolic derangements underlying congestion in HF more comprehensively than any single hormone. This hypothesized mechanism explains its incremental value over traditional markers.

Neurohormonal activation and renal–cardiac/metabolic perturbations are increasingly recognized as key components of HF congestion beyond natriuretic peptide elevation [35,36,37]. In this context, our findings suggest that SCS may capture a steroid-related vulnerability dimension that is less directly reflected by BNP. BNP is released primarily by cardiomyocytes in response to stretch and is a direct marker of ventricular wall stress [38]. Our correlation analysis found weak associations between BNP and most steroids, which supports the notion that SCS and BNP represent different but complementary dimensions of the pathophysiology in HF [39,40]. Furthermore, we observed a very weak correlation between SCS and high-sensitivity troponin I, whereas hs-TnI and BNP showed a modest correlation, suggesting that SCS captures pathophysiological information distinct from both myocardial stretch (BNP) and myocyte injury (hs-TnI). Specifically, the integration of cortisol, DHEAS, and pregnenolone in the SCS may reflect neurohormonal activation (hypothalamic-pituitary-adrenal axis dysregulation) and metabolic reprogramming (catabolic/anabolic imbalance), which are not directly assessed by natriuretic peptides or troponins. The incremental prognostic value of the SCS, particularly over the BNP model and over the hs-TnI model, further reinforces this notion. Moreover, unlike the CCS, which is derived from physical examination, the SCS can identify high-risk patients with “subclinical” congestion, whose persistent hemodynamic abnormalities may not be detected by routine examination but still contribute significantly to adverse outcomes [41,42,43]. From a clinical perspective, the SCS may guide intensified follow-up, optimization of diuretic and neurohormonal antagonist dosing, and closer hemodynamic monitoring for such high-risk patients during the vulnerable post-discharge phase. Therefore, as a blood-based multi-hormone marker, the SCS provides clinicians with an objective tool for quantitative assessment of the overall congestion burden.

The SCS is a continuous score and relatively simple to calculate, so has potential for adoption in clinical settings. This tool could be used for refinement of risk stratification when managing the vulnerable phase in patients who have been discharged [44]. Patients with a high SCS, even if symptomatically improved, should be considered high-risk, warranting intensified follow-up, consideration of more aggressive medical therapy (e.g., optimized diuresis, upward titration of neurohormonal antagonists), and possibly closer hemodynamic monitoring [45].

Subgroup analyses demonstrated that the prognostic value of the SCS was generally consistent regardless of age, sex, comorbidities, and cardiac functional status (all *p*-values for interaction were >0.05), confirming its generalizability and robustness as a risk stratification tool. Although higher point estimates for HRs were observed in patients with reduced LVEF, those who were male, and those with diabetes, the interaction tests did not reach statistical significance. Therefore, it is possible that these subgroup differences may result from random variation and that it is too early to conclude that the SCS has stronger prognostic value in these populations. Future studies in larger prospective cohorts are needed to confirm the predictive efficacy of the SCS across various clinical subgroups and to explore its value in guiding personalized treatment.

## 5. Limitations

This study has several limitations. First, all patients were enrolled from a single institution, and internal validation suggested mild overfitting of the SCS. Therefore, the generalizability of our findings requires confirmation in multicenter cohorts with diverse populations. Second, measurement of the SCS currently relies on liquid chromatography–tandem mass spectrometry, which may limit its accessibility in resource-limited settings. However, the cost and time required for this method are decreasing with technological advances. Third, validation cohort 2 included only 46 patients with paired samples, and the analyses of recongestion dynamics are therefore exploratory. Future studies should investigate the longitudinal trajectory of the SCS and its value in guiding adjustments to treatment. Finally, as an observational study, causality cannot be inferred from the observed associations. The proposed mechanistic interpretations should therefore be considered hypothesis-generating and require experimental validation.

## 6. Conclusion

This study constructed and validated a novel steroid-based congestion score, known as the SCS, which incorporates cortisol, DHEAS, and pregnenolone. This score is an independent predictor of adverse outcomes during the 90-day vulnerable phase and long-term follow-up in patients with AHF, offering incremental prognostic information beyond traditional clinical factors, BNP, and physical examination. The SCS is a biomarker that integrates neurohormonal and metabolic information and may provide a novel strategy for risk stratification and personalized management during the critical vulnerable post-discharge phase in HF.

## Data Availability

The datasets generated and analyzed during the current study are available from the corresponding author upon reasonable request.
