# Supplementary material for: Plasma Steroids as Biomarkers of Congestion and Predictors of Prognosis in Acute Heart Failure During the Vulnerable Phase
Source: Rev Cardiovasc Med. 2026 Jul 21;27(7):53378. doi: 10.31083/RCM53378 (PMC13419943; doi:10.31083/RCM53378)
Supplement: Supplementary file 1 [file 2153-8174-27-7-53378-s1.zip › Supplementary Material.pdf]

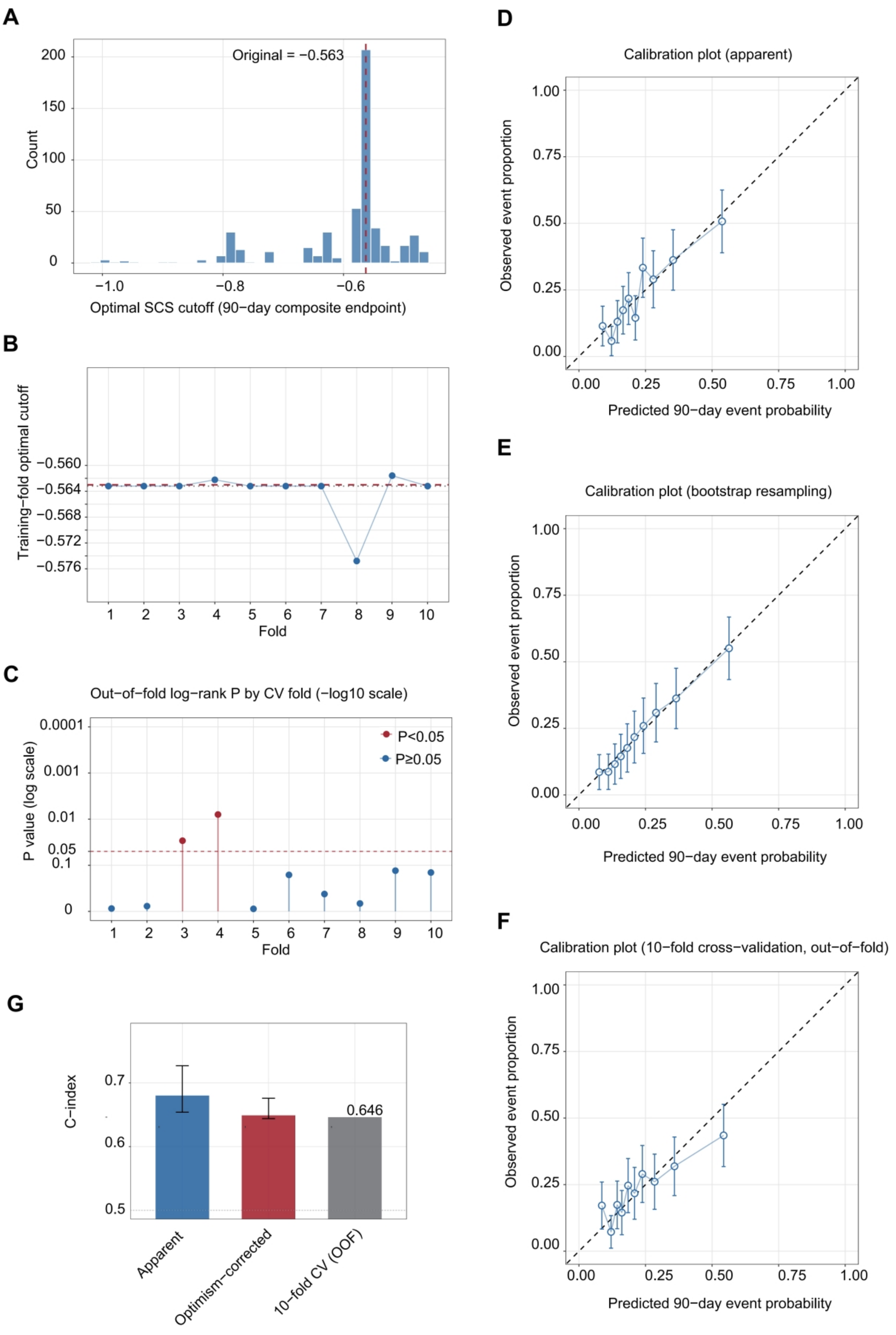

Supplementary Figure S1. Internal validation of the SCS model. Bootstrap and 10-fold cross-validation were performed in the discovery cohort (n=691). Panels (A) and (B) show cut-off stability. Panel (C) shows out-of-fold log-rank P values across folds. Panels (D–F) show calibration (apparent, bootstrap-corrected, and cross-validated). Panel (G) shows Harrell's C-index (apparent, optimism-corrected, and cross-validated).

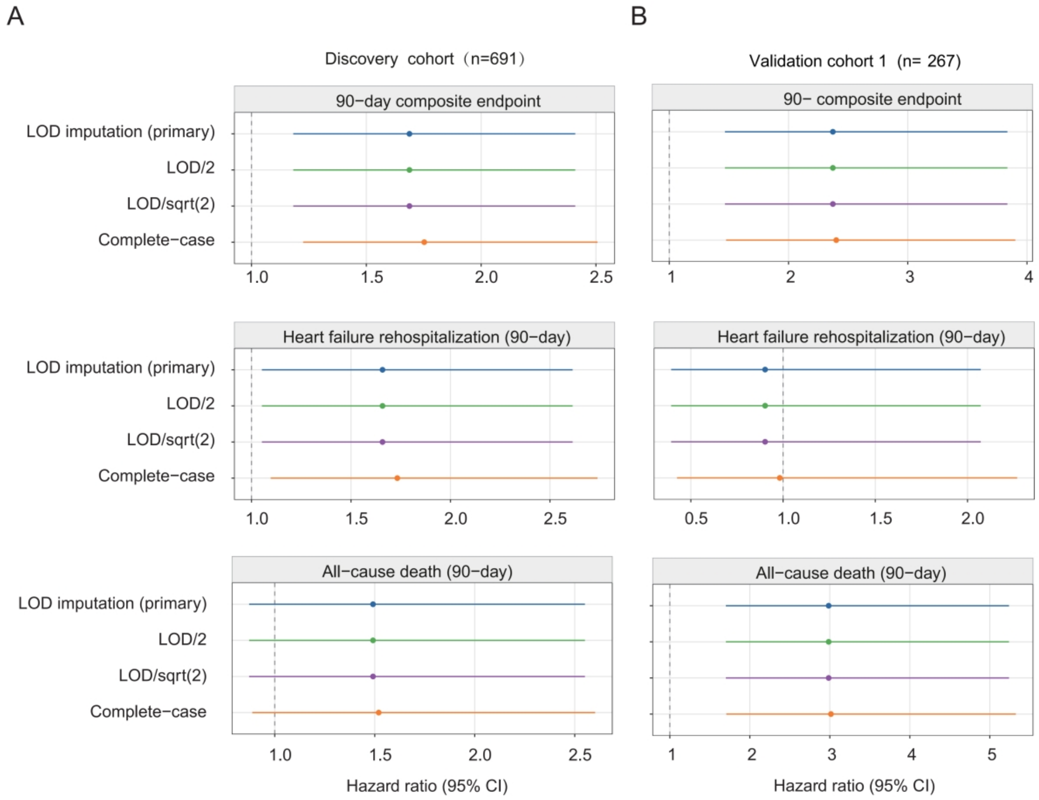

Supplementary Figure S2. Comparison of four LOD imputation strategies for SCS prognostic value. HRs (95% CI) are shown for the discovery cohort (n=691) and validation cohort 1 (n=267). The four strategies (LOD, LOD/2, LOD/√2, and complete-case) produced highly consistent effect estimates, indicating robustness of the primary imputation method.

Table S1. LOD and imputation proportions of 18 steroids in the discovery, validation 1, and validation 2 cohorts.

		Discovery (N=691)		Validation cohort 1 (N=267)		Validation cohort 2 (N=92)	
Steroid hormone	LOD (ng/mL)	N imputed	%	N imputed	%	N imputed	%
Pregnenolone	0.01	2	0.29	0	0	0	0
Progesterone	0.05	238	34.44	87	32.58	32	34.78
11-Deoxycorticosterone	0.02	143	20.69	75	28.09	20	21.74
17-OH progesterone	0.02	3	0.43	5	1.87	0	0
Corticosterone	0.02	12	1.74	15	5.62	4	4.35
11-Deoxycortisol	0.02	11	1.59	3	1.12	1	1.09
Cortisone	0.1	0	0	0	0	0	0
Cortisol	0.5	5	0.72	5	1.87	0	0
Aldosterone	0.002	2	0.29	1	0.37	1	1.09
Estrone	0.01	482	69.75	168	62.92	70	76.09
Estradiol	0.01	225	32.56	99	37.08	39	42.39
Estriol	0.01	531	76.85	206	77.15	77	83.7
17-OH pregnenolone	0.02	47	6.8	15	5.62	3	3.26
DHEAS	5	3	0.43	0	0	0	0
DHEA	0.05	80	11.58	32	11.99	10	10.87
Androstenedione	0.02	3	0.43	6	2.25	0	0
Testosterone	0.02	15	2.17	4	1.5	1	1.09
DHT	0.01	38	5.5	16	5.99	0	0

Abbreviations: LOD, limit of detection.

Table S2. Spearman Correlations between Steroid Hormones and Clinical Parameters in the Discovery Cohort.

	BMI		BNP		CCS		Cr		DBP		Heart rate		Hb		Na		SBP	
	r	P-value	r	P-value	r	P-value	r	P-value	r	P-value	r	P-value	r	P-value	r	P-value	r	P-value
DHEAS	0.08	0.032	-0.06	0.090	-0.13	<0.001	-0.04	0.292	0.01	0.694	0.05	0.170	0.05	0.223	-0.06	0.134	-0.00	0.922
Pregnenolone	0.01	0.709	-0.05	0.233	0.12	0.001	0.04	0.240	0.08	0.026	0.11	0.003	0.12	0.002	0.02	0.636	0.01	0.696
Cortisol	-0.10	0.007	0.09	0.024	0.11	0.004	0.16	<0.001	0.02	0.608	0.18	<0.001	-0.02	0.665	-0.11	0.003	-0.03	0.431
DHEA	-0.05	0.157	0.00	0.975	-0.07	0.062	-0.11	0.003	0.05	0.225	0.07	0.054	0.13	<0.001	0.02	0.601	-0.02	0.535
Estriol	0.10	0.012	0.01	0.737	0.06	0.105	0.04	0.329	0.05	0.166	0.08	0.035	0.10	0.006	-0.04	0.353	0.00	0.980
Testosterone	0.10	0.007	0.05	0.219	0.06	0.147	0.10	0.006	0.10	0.009	-0.04	0.329	0.36	<0.001	-0.01	0.811	0.03	0.398
Estradiol	0.04	0.260	-0.01	0.831	-0.05	0.223	-0.02	0.551	0.00	0.963	0.03	0.405	0.15	<0.001	-0.02	0.534	-0.06	0.139
17-OH pregnenolone	-0.03	0.370	-0.06	0.122	-0.04	0.263	-0.06	0.121	0.11	0.005	0.11	0.003	0.05	0.154	0.00	0.967	0.04	0.312
Androstenedione	-0.03	0.371	0.04	0.343	0.04	0.284	0.07	0.054	0.10	0.006	0.18	<0.001	0.02	0.634	-0.02	0.556	-0.02	0.675
Aldosterone	-0.04	0.266	0.08	0.037	-0.04	0.310	0.03	0.497	-0.05	0.184	0.08	0.034	0.09	0.025	-0.15	<0.001	-0.08	0.030
Estrone	-0.08	0.031	0.04	0.267	0.04	0.347	0.06	0.111	0.00	0.938	0.14	<0.001	-0.02	0.565	-0.09	0.024	-0.08	0.028
Progesterone	-0.07	0.057	0.03	0.444	0.04	0.353	0.07	0.058	0.05	0.219	0.17	<0.001	0.01	0.784	-0.12	0.002	-0.05	0.223
Cortisone	-0.01	0.696	-0.04	0.300	-0.03	0.489	-0.22	<0.001	0.12	0.002	0.05	0.156	0.30	<0.001	0.09	0.025	-0.10	0.009
Corticosterone	-0.13	<0.001	0.10	0.006	0.02	0.578	0.02	0.597	-0.01	0.863	0.16	<0.001	0.03	0.360	-0.07	0.087	-0.08	0.027
17-OH progesterone	0.04	0.253	-0.04	0.357	-0.02	0.587	0.02	0.620	0.10	0.012	0.08	0.044	0.14	<0.001	-0.01	0.856	0.06	0.119
DHT	0.06	0.106	-0.01	0.778	-0.02	0.627	-0.02	0.560	0.05	0.163	-0.06	0.115	0.33	<0.001	0.04	0.325	0.00	0.953
DOC	-0.11	0.004	0.05	0.195	-0.01	0.779	0.02	0.566	0.03	0.507	0.22	<0.001	-0.01	0.733	-0.10	0.009	-0.05	0.166
11-Deoxycortisol	-0.08	0.035	0.06	0.142	-0.00	0.913	0.06	0.095	0.02	0.636	0.16	<0.001	-0.04	0.308	0.00	0.987	-0.03	0.485

Table S3. Cox Regression Analysis for the Association of SCS with 90-Day Outcomes in the Discovery Cohort.

Endpoint	HR (95% CI)	P Value	Adjusted HR (95% CI)	P Value	Adjusted HR (95% CI)	P Value	Adjusted HR (95% CI)	P Value
	model 1		model 2		model 3		model 4	
Composite Endpoint								
SCS（per 1 SD）	1.22 (1.05-1.42)	0.009	1.23 (1.05-1.44)	0.010	1.12 (0.95-1.32)	0.187	1.12 (0.95-1.33)	0.179
SCS < -0.563	Ref.		Ref.					
SCS ≥ -0.563	1.95 (1.39-2.73)	<0.001	2.02 (1.42-2.88)	<0.001	1.69 (1.18-2.41)	0.004	1.68 (1.17-2.41)	0.005
All-cause Mortality								
SCS（per 1 SD）	1.15 (0.91-1.44)	0.242	1.15 (0.92-1.45)	0.220	1.01 (0.78-1.31)	0.932	1.02 (0.79-1.32)	0.862
SCS < -0.563	Ref.		Ref.					
SCS ≥ -0.563	1.89 (1.15-3.12)	0.013	1.96 (1.16-3.31)	0.011	1.49 (0.87-2.55)	0.144	1.60 (0.93-2.75)	0.091
Heart Failure Rehospitalization								
SCS（per 1 SD）	1.28 (1.06-1.54)	0.01	1.27 (1.04-1.54)	0.017	1.18 (0.96-1.45)	0.114	1.18 (0.96-1.46)	0.11
SCS < -0.563	Ref.		Ref.					
SCS ≥ -0.563	1.96 (1.27-3.01)	0.002	1.95 (1.24-3.07)	0.004	1.66 (1.05-2.61)	0.029	1.62 (1.02-2.56)	0.041

Table S4. Subgroup Analysis for the Association of SCS with 90-Day Outcomes in Validation Cohort 1

	Composite Endpoint		Mortality	
	HR (95% CI)	P interaction	HR (95% CI)	P interaction
All Patients	1.39 (1.14-1.70)		1.64 (1.32-2.04)	
Age		0.235		0.623
<60 y	1.63 (1.06-2.51)		2.40 (1.53-3.77)	
≥60 y	1.50 (1.19-1.90)		1.63 (1.25-2.12)	
Sex		0.549		0.466
Female	1.41 (1.02-1.96)		1.68 (1.17-2.42)	
Male	1.43 (1.11-1.84)		1.64 (1.23-2.18)	
Hypertension		0.082		0.142
No	1.93 (1.29-2.91)		2.52 (1.55-4.09)	
Yes	1.27 (0.98-1.66)		1.48 (1.11-1.98)	
Diabetes		0.341		0.228
No	1.60 (1.18-2.16)		1.95 (1.39-2.75)	
Yes	1.29 (0.97-1.71)		1.57 (1.15-2.14)	
LVEF		0.220		0.459
<50%	1.53 (1.21-1.93)		1.80 (1.39-2.32)	
≥50%	1.19 (0.68-2.06)		1.47 (0.76-2.81)	
Diuretics		0.094		0.071
No	1.40 (0.99-1.99)		1.98 (1.27-3.09)	
Yes	1.74 (1.29-2.33)		2.14 (1.54-2.96)	

Supplementary Table S5. Comparison of four LOD imputation strategies for SCS prognostic value.

**Discovery cohort（n=691）**	**HR (95% CI)**	**P value**
**90-day composite endpoint**		
LOD imputation (primary)	1.69 (1.18-2.41)	0.004
LOD/2	1.69 (1.18-2.41)	0.004
LOD/sqrt(2)	1.69 (1.18-2.41)	0.004
Complete-case	1.75 (1.22-2.51)	0.002
**All-cause death (90-day)**		
LOD imputation (primary)	1.49 (0.87-2.55)	0.144
LOD/2	1.49 (0.87-2.55)	0.144
LOD/sqrt(2)	1.49 (0.87-2.55)	0.144
Complete-case	1.52 (0.89-2.60)	0.127
**Heart failure rehospitalization (90-day)**		
LOD imputation (primary)	1.66 (1.05-2.61)	0.029
LOD/2	1.66 (1.05-2.61)	0.029
LOD/sqrt(2)	1.66 (1.05-2.61)	0.029
Complete-case	1.73 (1.10-2.74)	0.019
**Validation cohort 1（n=267）**		
**90-day composite endpoint**		
LOD imputation (primary)	2.37 (1.47-3.84)	<0.001
LOD/2	2.37 (1.47-3.84)	<0.001
LOD/sqrt(2)	2.37 (1.47-3.84)	<0.001
Complete-case	2.40 (1.48-3.90)	<0.001
**All-cause death (90-day)**		
LOD imputation (primary)	2.99 (1.70-5.24)	<0.001
LOD/2	2.99 (1.70-5.24)	<0.001
LOD/sqrt(2)	2.99 (1.70-5.24)	<0.001
Complete-case	3.01 (1.71-5.33)	<0.001
**Heart failure rehospitalization (90-day)**		
LOD imputation (primary)	0.90 (0.39-2.07)	0.809
LOD/2	0.90 (0.39-2.07)	0.809
LOD/sqrt(2)	0.90 (0.39-2.07)	0.809
Complete-case	0.98 (0.43-2.27)	0.966

HR, hazard ratio; CI, confidence interval; LOD, limit of detection; LOD/2, imputation with half the LOD value; LOD/√2, imputation with LOD divided by the square root of 2; complete-case, analysis excluding all samples with values below the LOD.

Table S6. Subgroup Analysis for the Association of SCS with Long-Term Outcomes (1200 Days) in Validation Cohort 1.

	Composite Endpoint		Mortality	
	HR (95% CI)	P interaction	HR (95% CI)	P interaction
All Patients	1.96 (1.33-2.90)		2.02 (1.28-3.18)	
Age	2.10 (1.43-3.07)	0.181	2.33 (1.49-3.63)	0.212
<60 y	6.05 (1.99-18.42)		9.33 (2.65-32.80)	
≥60 y	1.81 (1.18-2.78)		1.91 (1.15-3.16)	
Sex	1.96 (1.32-2.89)	0.598	2.03 (1.29-3.19)	0.881
Female	1.54 (0.85-2.80)		1.86 (0.94-3.70)	
Male	2.14 (1.22-3.75)		2.79 (1.40-5.55)	
Hypertension	1.97 (1.34-2.90)	0.404	2.04 (1.29-3.21)	0.929
No	3.14 (1.54-6.43)		2.78 (1.24-6.21)	
Yes	1.72 (1.05-2.81)		2.05 (1.16-3.63)	
Diabetes	1.87 (1.27-2.73)	0.951	2.01 (1.28-3.16)	0.419
No	1.91 (1.13-3.23)		2.65 (1.45-4.83)	
Yes	2.24 (1.17-4.28)		2.12 (0.99-4.55)	
LVEF	1.96 (1.33-2.90)	0.036	2.02 (1.28-3.18)	0.054
<50%	2.76 (1.72-4.42)		2.93 (1.68-5.11)	
≥50%	0.66 (0.24-1.80)		0.63 (0.19-2.06)	
Diuretics	1.98 (1.35-2.91)	0.135	2.04 (1.30-3.20)	0.117
No	1.26 (0.57-2.76)		1.68 (0.68-4.15)	
Yes	2.44 (1.51-3.94)		2.47 (1.39-4.36)	

Table S7. Association of baseline SCS with 90-day congestion change in Validation Cohort 2 (n=46 paired patients)

**Outcome (**Recongestion**)**	**Odds Ratio (95% CI)**
SCS- High vs SCS- Low	1.57 (0.07-35.03)
SCS- High vs SCS- Mid	2.02 (0.30-13.83)
